# Molecular signaling pathways shaping astrocyte–microglia crosstalk in health and disease

**DOI:** 10.3389/fnmol.2026.1828420

**Published:** 2026-08-06

**Authors:** Pilar Argente-Arizón, Belén Monge-Ochoa

**Affiliations:** Facultad de Ciencias de la Salud, Universidad San Jorge, Zaragoza, Spain

**Keywords:** astrocytes, glial crosstalk, microglia, molecular signaling pathways, neuroinflammation, neuroprotection

## Abstract

While neuron–glia communication has been extensively investigated, the molecular dialogue between non-neuronal cells, particularly astrocytes and microglia, remains comparatively less explored. This bidirectional crosstalk plays a central role in maintaining central nervous system (CNS) homeostasis, coordinating responses to injury, and shaping neuroinflammatory dynamics. Astrocyte–microglia communication is mediated by a complex network of secreted factors and intracellular signaling pathways that shape glial activation states and functional outcomes. Among the best-characterized pathways, NF-κB, JAK/STAT3, MAPK/ERK, and Smad2/3 regulate the balance between pro-inflammatory and reparative responses and influence whether glial signaling promotes neurotoxicity or neuroprotection. In this mini-review, we summarize the molecular mechanisms underlying astrocyte–microglia crosstalk in physiological and pathological contexts, with particular emphasis on the secreted mediators that dynamically reprogram glial signaling networks. We also highlight how emerging experimental platforms, including human induced pluripotent stem cell-derived glia, brain organoids, and organ-on-chip systems, are helping to uncover human-specific and disease-relevant features of glial interaction. A better understanding of astrocyte–microglia molecular crosstalk may help define the signaling programs that sustain chronic neuroinflammation and neurodegeneration. Such insight could support the development of strategies aimed at restoring glial homeostasis and limiting disease progression.

## 1 Introduction

Glial cells, once historically conceptualized as passive “glue-like” elements of the central nervous system (CNS), are now recognized as a diverse and functionally active group that includes astrocytes, oligodendrocytes, microglia, ependymal cells, and tanycytes. Beyond their classical supportive roles, glial cells actively regulate CNS development, homeostasis, and plasticity, and contribute to the pathophysiology of neurological disease (Von Bartheld et al., 2016; Lee et al., 2022). While neuron–glia interactions have been extensively characterized, reflecting a longstanding emphasis on neuron-centered models of CNS function (Hu and Tao, 2024), communication among non-neuronal cells themselves has received comparatively less integrative attention and is the focus of this review.

Among glial populations, astrocytes and microglia occupy central and complementary positions in regulating brain homeostasis and immune surveillance. Astrocytes constitute a major glial population in the CNS and perform a wide array of functions including maintenance of ionic and neurotransmitter homeostasis, metabolic support to neurons, modulation of synaptic transmission, and preservation of blood–brain barrier (BBB) integrity (Anderson and Nedergaard, 2003; Clarke and Barres, 2013). Their extensive morphological complexity and strategic positioning at synaptic and vascular interfaces enable astrocytes to influence neuronal networks at both local and global levels (Bushong et al., 2002; Machado-Santos et al., 2024). Importantly, astrocytes also engage in dynamic communication with microglia, extending their influence beyond neuron-centered signaling paradigms.

Microglia, the resident innate immune cells of the CNS, are uniquely equipped to sense alterations in the neural microenvironment. Under homeostatic conditions, they continuously survey the parenchyma through highly motile processes, while upon injury or infection they rapidly transition to activated states characterized by migration, phagocytosis, and cytokine production (Nimmerjahn et al., 2005; Baufeld et al., 2018). In addition to their immune functions, microglia play essential roles during neurodevelopment by supporting neuronal survival, synaptic refinement, and circuit maturation (Cunningham et al., 2013).

The reciprocal communication between astrocytes and microglia constitutes a highly dynamic and context-dependent signaling network that is increasingly recognized as fundamental to CNS physiology and pathology. These interactions are mediated through multiple mechanisms, including direct cell–cell contact, secretion of soluble factors such as cytokines, chemokines, and growth factors, purinergic signaling, and the exchange of extracellular vesicles (Delpech et al., 2019; Matejuk and Ransohoff, 2020; Rostami et al., 2021). The nature and outcome of astrocyte–microglia crosstalk vary across developmental stages, during homeostatic maintenance, and in response to injury or neurodegenerative processes.

Despite growing interest in glial interactions, the molecular logic by which astrocytes and microglia integrate extracellular cues into coordinated intracellular signaling responses remains incompletely understood, particularly in human-relevant contexts. In this mini-review, we focus on the signaling pathways and secreted mediators that shape astrocyte–microglia crosstalk in health and disease, with special emphasis on how these interactions are reprogrammed in neurodegenerative settings and how emerging human-based models may help bridge the gap between experimental systems and disease-relevant biology.

## 2 Mechanisms of astrocyte-microglia communication

Astrocyte–microglia communication is a dynamic and context-dependent process that plays a central role in CNS homeostasis and becomes especially relevant under pathological conditions. Rather than acting through a single route, these glial populations communicate through multiple complementary mechanisms that allow them to coordinate responses to changes in the neural microenvironment and shape neuronal function and survival.

Under physiological conditions, astrocytes and microglia cooperate to support synaptic function, maintain immune surveillance, preserve BBB integrity, and regulate the extracellular environment (Machado-Santos et al., 2024). Astrocytes contribute to ion balance, neurotransmitter clearance, and metabolic support, whereas microglia continuously survey the parenchyma for subtle alterations in tissue status. Together, these cells help maintain a stable and adaptive CNS milieu. In addition, astrocytes contribute to neuroprotection during oxidative stress by supplying antioxidant molecules such as glutathione, ascorbate, and vitamin E (Makar et al., 1994; Pérez-Sala and Pajares, 2023; Yoshioka et al., 2021), whereas astrocytes and microglia can support neuronal resilience through the release of trophic and anti-inflammatory mediators, including growth factors and cytokines that favor tissue repair (Cunningham et al., 2013; Kuno et al., 2006; Rizzi et al., 2018). Importantly, astrocytes and microglia do not respond to CNS perturbations with identical temporal dynamics. Microglia are highly motile immune-surveillance cells that can rapidly extend their processes and modify their interactions with surrounding structures within seconds to minutes after injury or changes in neuronal activity (Nimmerjahn et al., 2005). In contrast, astrocytic morphological remodeling generally develops over hours to days, although transcriptional, metabolic, and cytokine-related responses can occur considerably earlier. These distinct temporal profiles are particularly relevant when distinguishing primary and secondary glial responses after injury or disease-associated insults, and they influence the transition from adaptive neuroprotective signaling to maladaptive neuroinflammatory states.

**FIGURE 1:**
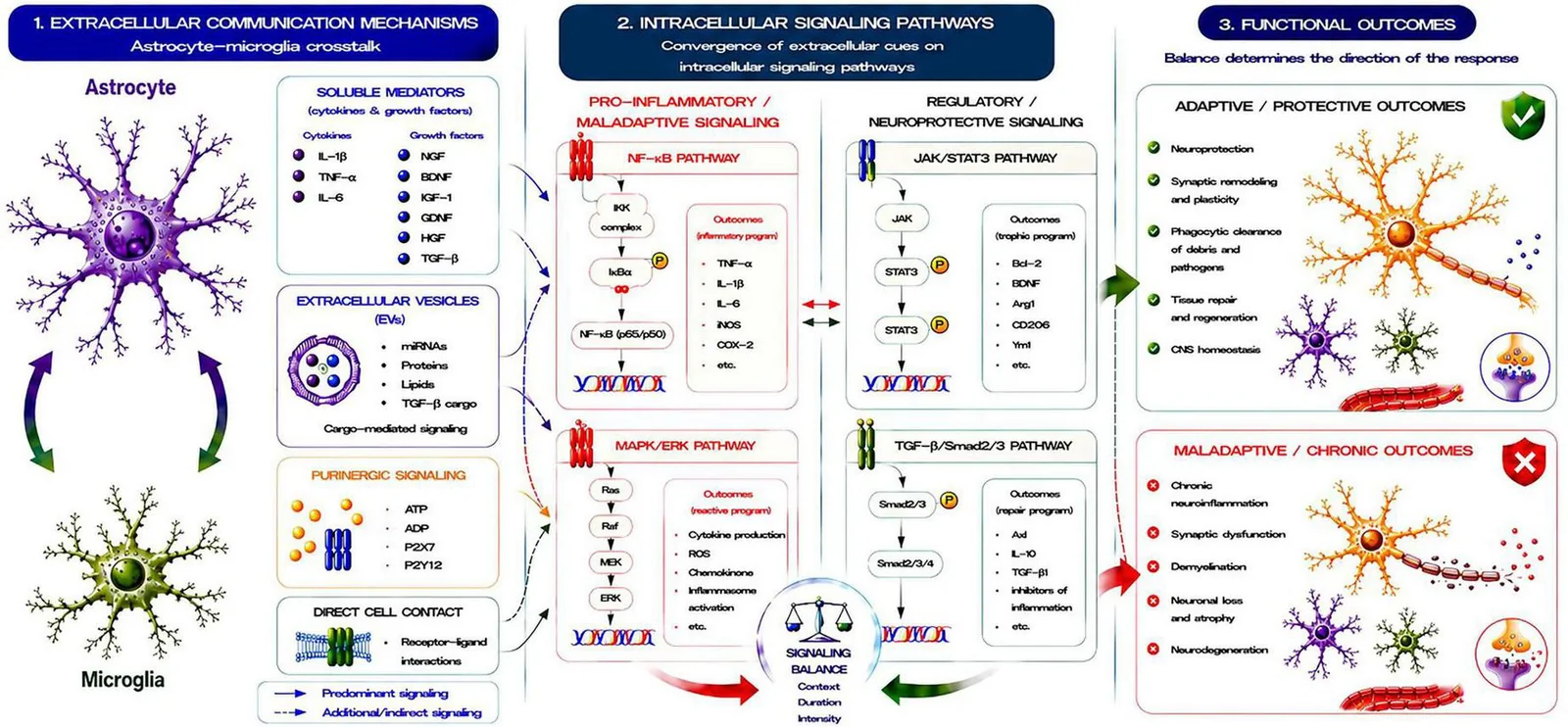
Molecular signaling pathways shaping astrocyte-microglia crosstalk and functional outcomes in astrocytes and microglia communicate through multiple extracellular mechanisms, including soluble mediators, extracellular vesicles (EVs), purinergic signaling, and direct cell-cell interactions. (e.g., IL-1β, TNF-α, and IL-6), growth factors (e.g., NGF, BDNF, IGF-1, GDNF, HGF, and TGF-B), and EV cargo containing proteins, lipids, and regulatory nucleic acids function as extracellular cues that converge on intracellular signaling pathways in astrocytes and microglia. These signaling programs include predominantly pro-inflammatory pathways, such as NF-kB and MAPK/ERK, as well as regulatory and neuroprotective pathways, including JAK/STAT3 and TGF-B/Smad2/3. The balance, intensity, and temporal coordination of these signaling networks determine whether astrocyte-microglia crosstalk promotes adaptive responses, including neuroprotection, synaptic remodeling, phagocytic clearance, and tissue repair, or maladaptive responses associated with chronic neuroinflammation, synaptic dysfunction, demyelination, neuronal loss, and neurodegeneration. IL-1β, interleukin-1 beta; TNF-α, tumor necrosis factor alpha; IL-6, interleukin-6; NGF, nerve growth factor; BDNF, brain-derived neurotrophic factor; IGF-1, insulin-like growth factor-1; GDNF, glial cell line-derived neurotrophic factor; HGF, hepatocyte growth factor; TGF-B, transforming growth factor beta; ATP, adenosine triphosphate; ADP, adenosine diphosphate; P2X7, purinergic receptor P2X7; P2Y12, purinergic receptor P2Y12; IKK, IKB kinase; IkBα, inhibitor of KB alpha; NF-κB, nuclear factor kappa-light-chain-enhancer of activated B cells; MAPK, mitogen-activated protein kinase; ERK, extracellular signal-regulated kinase; JAK, Janus kinase; STAT3, signal transducer and activator of transcription 3; Smad2/3, mothers against decapentaplegic homolog 2/3; Smad complex.

At the mechanistic level, astrocyte–microglia communication occurs through three main routes (Figure 1). The first involves direct cell–cell contact, mediated by adhesion molecules and junctional proteins. Molecules such as ICAM-1 and VCAM-1 can be upregulated in inflammatory contexts and facilitate intercellular interactions associated with immune cell recruitment and BBB dysfunction (Spampinato et al., 2019). In parallel, connexin-based channels, including CX43, may contribute to the exchange of ions and small metabolites between glial cells, although their precise contribution to astrocyte–microglia crosstalk remains incompletely defined (Abudara et al., 2015).

The second route is mediated by soluble factors, including cytokines, chemokines, and growth factors. These signals are among the most important regulators of bidirectional glial communication. Astrocyte-derived IL-33 can promote microglial synaptic engulfment during development (Vainchtein et al., 2018), whereas microglial IL-1β can induce reactive astrogliosis and amplify inflammatory signaling (Smith et al., 2019; Hyvärinen et al., 2019; Pamies et al., 2021). Other mediators, including TNF-α, IL-6, CXCL10, can promote or sustain glial activation in a context-dependent manner and contribute to chronic inflammatory states depending on timing, concentration, and disease setting. Activated microglia-derived cytokines such as TNF-α and IL-6 have been shown to induce astrocytic inflammatory phenotypes, whereas inflammatory niches can further amplify CXCL10 production in glial cultures (Smith et al., 2019; Hyvärinen et al., 2019; Pamies et al., 2021; Wang et al., 2024). In parallel, growth factors such as IGF-1 and GDNF, may support adaptive responses and glial resilience under specific conditions, contributing to repair-associated phenotypes and modulation of inflammatory responses (Suh et al., 2013; Toyomoto et al., 2005).

The third mechanism involves extracellular vesicles (EVs), including exosomes and microvesicles. These vesicles transport proteins, lipids, and nucleic acids that can alter gene expression and functional responses in recipient cells. By transferring regulatory microRNAs, mRNAs, proteins, and lipid mediators to recipient glial cells, EV cargo can reshape transcriptional programs, inflammatory responsiveness, and repair-associated signaling pathways, thereby influencing neuroinflammation, synaptic remodeling, and neuronal survival. Astrocyte and microglia-derived extracellular vesicles have been implicated in the regulation of neuroinflammation, synaptic remodeling, and neuronal survival under both physiological and pathological conditions (Delpech et al., 2019; Rostami et al., 2021; Van Niel et al., 2022). By transferring bioactive cargo between cells, extracellular vesicles provide an additional layer of communication that extends beyond classical ligand–receptor signaling (Gao et al., 2023).

These communication routes converge on a limited number of intracellular signaling pathways that shape glial phenotypes and determine whether the overall outcome is protective or harmful. NF-κB is a key regulator of pro-inflammatory responses and promotes the expression of cytokines such as IL-1β, TNF-α, and IL-6. In contrast, JAK/STAT3 signaling is often associated with reactive but potentially reparative astrocytic programs, including the modulation of inflammatory mediators and stress-response genes (Toral-Rios et al., 2020). Smad2/3 downstream of TGF-β contributes to immunoregulatory and tissue-repair programs (Butovsky et al., 2014), while MAPK/ERK acts as an integrative hub that translates extracellular cues into changes in glial plasticity, activation, and survival (Baumann et al., 2024; Kang et al., 2025; Zhang Z. et al., 2025).

Collectively, these molecular routes form a signaling framework through which astrocytes and microglia integrate environmental cues and coordinate their responses in health and disease. The balance between neuroprotective and neurotoxic outcomes depends on the intensity, duration, and cellular context of these signals. Understanding how these communication mechanisms are organized provides the basis for analyzing the specific secreted mediators that shape astrocyte–microglia crosstalk in the next section, as well as their contribution to neuroinflammatory and neurodegenerative processes (Kwon and Koh, 2020; Wu and Eisel, 2023; Sochocka et al., 2017).

## 3 Secreted molecular mediators shaping astrocyte–microglia signaling

Astrocyte–microglia communication is critically shaped by a diverse repertoire of secreted molecular mediators that act as extracellular inputs to intracellular signaling pathways. Rather than functioning as isolated signals, these mediators dynamically regulate glial activation states, reciprocal responsiveness and fine-tune the balance between homeostatic and reactive phenotypes in a context-dependent manner across physiological conditions, injury, and disease.

Among these mediators, growth factors occupy a central place in coordinating glial crosstalk and shaping glial activating states. Brain-derived neurotrophic factor (BDNF), produced by both astrocytes and microglia, supports neuronal survival and synaptic plasticity while promoting microglial phenotypes associated with neuroprotection and tissue repair (Saha et al., 2006; Komori et al., 2024) . In parallel, microglia-derived insulin-like growth factor 1 (IGF-1) contributes to glial homeostasis and supports neuronal resilience, (Zhang et al., 2026), whereas IGF-1 signaling can also modulate astrocytic inflammatory responses under stress conditions (Pinto-Benito et al., 2022). Additional trophic mediators, including glial cell line-derived neurotrophic factor (GDNF) and hepatocyte growth factor (HGF), further contribute to adaptative glial responses (Toyomoto et al., 2005). Recent studies indicate that reactive astrocytes can release GDNF to promote neuronal survival and functional recovery (Zhang Z. et al., 2025) whereas HGF signaling attenuates astrocytic reactivity and reduces GFAP expression under injury-like conditions (Hong et al., 2021). Although these trophic mediators act through distinct receptor systems, they converge on intracellular signaling networks associated with cell survival, plasticity, and repair, including MAPK/ERK, PI3K/Akt, and context-dependent STAT3 signaling (Hong et al., 2021; Ulloa et al., 2024).

Nerve growth factor (NGF) is another important mediator of astrocyte–microglia communication. Astrocytes can upregulate NGF in response to inflammatory cues, while NGF directly steers microglia toward a neuroprotective and anti-inflammatory phenotype, supporting reciprocal glial regulation under stress conditions (Rizzi et al., 2018; Kuno et al., 2006). Collectively, these trophic mediators function as key modulators of astrocyte–microglia communication, integrating inflammatory and neuroprotective signaling programs across physiological and pathological contexts.

In addition to classical growth factors, cytokines and vascular mediators released by microglia further shape astrocytic behavior. Microglia-derived transforming growth factor-β (TGF-β) acts as a key immunoregulatory signal that constrains excessive astrocytic activation and promotes tissue repair (Taylor et al., 2017; Zhang et al., 2020). Notably, extracellular vesicles released from hypoxia-preconditioned microglia and containing EV-associated TGF-β1 can modulate astrocyte function and enhance neurovascular repair through activation of the TGF-β/Smad2/3 pathway, highlighting the importance of paracrine signaling modes in glial communication (Zhang et al., 2021; Butovsky et al., 2014).

Beyond protein mediators, purinergic signaling constitutes a rapid and highly dynamic mechanism of astrocyte–microglia communication. Astrocytes release ATP and ADP into the extracellular space through both Ca^2 +^-dependent exocytosis and Ca^2 +^-independent mechanisms, including stretch-activated channels and P2 × 7 receptor pores (Xiong et al., 2018; Li et al., 2025). These nucleotides engage purinergic receptors expressed on microglia, such as ionotropic P2X and metabotropic P2Y receptors, thereby regulating microglial motility, chemotaxis, cytokine release, and phagocytic activity (Bałkowiec-Iskra et al., 2024). Activation of microglial P2 × 7 receptors by ATP can promote the release of extracellular vesicles and pro-inflammatory mediators, whereas signaling through P2Y receptors, particularly P2Y12 and P2Y1, has been associated with surveillance functions and anti-inflammatory responses (Shinozaki et al., 2014; Pósfai et al., 2026).

These secreted mediators converge on intracellular signaling pathways, including NF-κB, JAK/STAT3, Smad2/3, and MAPK/ERK cascades, which ultimately determine glial activation states and functional outcomes ranging from neuroprotection to neurotoxicity (Ulloa et al., 2024; Wang et al., 2026). By modulating the intensity, duration, and spatial organization of these signaling events, extracellular growth factors, cytokines, and purinergic signals critically shape astrocyte–microglia crosstalk. Dysregulation of these mediator-driven signaling networks emerges as a key determinant of maladaptive glial responses, linking altered intercellular communication to chronic neuroinflammation and neurodegenerative disease.

Recent studies using experimental models further highlight the context-dependent nature of glial signaling and the dynamic reprogramming of astrocyte–microglia communication in response to environmental cues and pathological stimuli. *In vivo* evidence shows that microglia can instruct astrocyte-synapse interactions through Wnt signaling in an activity-dependent manner (Faust et al., 2025), whereas astrocyte-derived signals such as Hevin can shape microglial phagocytic responses during circuit refinement and disease-relevant remodeling (Ramirez et al., 2026).

## 4 Astrocyte–microglia crosstalk in neuroinflammatory and neurodegenerative disorders

In pathological contexts, astrocyte–microglia communication becomes profoundly altered, shifting from homeostatic signaling toward sustained neuroinflammatory responses. Although neuroinflammation initially represents a protective mechanism, its persistence can drive chronic inflammation, synaptic dysfunction and progressive neuronal injury. In neurodegenerative conditions, sustained activation of NF-κB, reinforces pro-inflammatory cytokine production and feed-forward inflammatory loops (Frakes et al., 2014; Rocha et al., 2023), whereas altered JAK/STAT3, MAPK/ERK and TGF-β/Smad signaling has been associated with reactive astrocyte phenotypes, microglial dysfunction, and impaired neuroprotective responses in disease models (Luo, 2022; Lee et al., 2023; Chen et al., 2023; O’Callaghan et al., 2014).

This dysregulation is particularly evident in Alzheimer’s disease (AD) and multiple sclerosis (MS), bidirectional communication between astrocytes and microglia becomes maladaptive and further amplifies inflammatory responses. In AD and related neurodegenerative settings, activated microglia can induce A1-like astrocytes through IL1-β, TNF-α, and C1q, a phenotype associated with loss of neuroprotective functions and neuronal injury (Liddelow et al., 2017; Poulot-Becq-Giraudon et al., 2026). A complementary mechanism involves the complement C3–C3aR axis, which mediates astrocyte–microglia communication and contributes to synaptic dysfunction and neurodegeneration. Recent findings indicate that astrocyte-derived complement C3 facilitates microglial phagocytosis of synapses, highlighting complement signaling as a key driver of pathological glial crosstalk in neurodegenerative conditions (Wei et al., 2021; Zhang H. et al., 2025).

Beyond complement-mediated signaling, several additional molecular pathways have been implicated in disease-associated astrocyte–microglia crosstalk. In Parkinson’s disease models, dysregulation of the C3–C3aR pathway has been linked to enhanced astrocyte–microglia communication and dopaminergic neuron degeneration (Zhang et al., 2023). In neuropathic pain models, paeoniflorin modulates astrocyte–microglia interaction through HSP90AA1/HMGB1 signaling, reducing inflammatory responses and neuronal apoptosis (Luo et al., 2024). Moreover, experimentally induced CNTN1 overexpression in the hippocampus triggers microglial activation and subsequent astrocytic reactivity, impairing synaptic plasticity and causing cognitive deficits (Li et al., 2023). This suggests that CNTN1 acts as an upstream disease-associated perturbation that can initiate glial activation and secondarily amplify astrocyte–microglia inflammatory crosstalk. In addition, astrocyte-derived soluble ANPEP can activate microglia and promote neuroinflammation through the brain renin–angiotensin system, highlighting another route by which astrocytes can shape microglial responses in disease (Kim et al., 2022).

These disease-associated interactions are better understood in the context of the broader activation states adopted by both cell types after CNS injury, infection, or exposure to toxic stimuli. Although the terms “activation” and “reactivity” are sometimes used interchangeably, they refer to partially distinct concepts in glial biology. Microglial activation generally describes the transition from surveillant homeostatic states toward altered immune, phagocytic, metabolic, and secretory programs in response to injury, infection, or disease-associated cues. By contrast, astrocyte reactivity refers to stimulus-dependent changes in astrocytic morphology, gene expression, metabolism, inflammatory signaling, and homeostatic support functions. Both processes are highly context-dependent and should be understood as dynamic state transitions rather than uniform or binary phenotypes. Microglial activation typically precedes astrocyte reactivity and serves as a key trigger for the emergence of reactive astrocytic programs. This sequence also reflects distinct temporal scales of glial responses: microglial processes can rapidly reorganize within seconds to minutes following injury or altered neuronal activity, allowing microglia to act as early sensors of tissue perturbation (Nimmerjahn et al., 2005), whereas astrocytic morphological remodeling generally develops over hours to days, although transcriptional and secretory responses can occur earlier (Lawrence et al., 2023). These temporal differences are relevant for distinguishing primary and secondary glial responses and may influence the transition from adaptive neuroprotective signaling to chronic maladaptive neuroinflammation. From a resting state, microglia respond to environmental signals by adopting an amoeboid morphology with enlarged soma and retracted processes, migrating to sites of injury to remove pathogens and cellular debris (Lawrence et al., 2023). In parallel, pro-inflammatory microglia produce cytokines such as IL-1β, IL-6, and TNF-α, as well as reactive species, whereas anti-inflammatory microglia support repair through cytokines such as IL-4 and IL-10 and through trophic factors such as BDNF and IGF-1 (Li et al., 2023; Badia-Soteras et al., 2025).

Astrocyte reactivity follows this microglial shift and can adopt pro-inflammatory or more reparative programs depending on the surrounding cues. A1-like astrocytes are induced by microglia-derived signals such as IL-1α, TNF-α, and C1q, and are associated with loss of homeostatic functions and promotion of neuronal death (Octeau et al., 2018; Lawrence et al., 2025). By contrast, more reparative astrocytic states can support neuronal survival and tissue repair. However, the A1/A2 dichotomy represents a simplified framework, because astrocyte reactivity encompasses a much broader spectrum of functional states. For clarity, Table 1 summarizes prototypical features historically associated with pro-inflammatory/neurotoxic and reparative/neuroprotective glial states, while emphasizing that these categories should be interpreted as heuristic reference points rather than discrete or fixed phenotypes. Taken together, these findings indicate that astrocyte-microglia crosstalk is not merely a bystander feature of pathology but a dynamic determinant of whether glial responses remain protective or become self-sustaining and harmful. Understanding how these interactions are shaped by disease context is therefore crucial for identifying therapeutic strategies that promote neuroprotection while limiting neurodegeneration.

**TABLE 1:** Prototypical features historically associated with neuroinflammatory and neuroprotective astrocyte and microglial states.

Feature	A1-like astrocytes (pro-inflammatory)	A2-like astrocytes (neuroprotective)	M1-like microglia (pro-inflammatory)	M2-like microglia (neuroprotective)	References
Inducing stimuli	IL-1α, TNF-α, C1q (from activated microglia)	Tissue injury, ischemia and regenerative cues	Lipopolysaccharide (LPS), IFN-γ, TNF-α, IL-1β	IL-4, IL-10, IL-13, TGF-β	Liddelow et al., 2017; Lawrence et al., 2023
Morphological features	Hypertrophy with thickened and disorganized processes	Mild hypertrophy with preserved morphology	Amoeboid shape with enlarged soma and retracted processes	Intermediate or ramified morphology with elongated processes	Octeau et al., 2018; Lawrence et al., 2023
Cytokine profile	IL-1β, TNF-α, IL-6, complement component C3	IL-10, TGF-β, neuroprotective cytokines	IL-1β, IL-6, TNF-α, CXCL10	IL-10, TGF-β, IL-4, IL-13	Liddelow et al., 2017; Tang and Le, 2016; Kwon and Koh, 2020
Molecular mediators and signaling pathways	IL-1β, TNF-α, C1q, nitric oxide (NO); NF-κB signaling	IL-10, TGF-β, BDNF, GDNF; STAT3-mediated signaling	IL-1β, IL-6, TNF-α, NO, ROS, PGE2; NF-κB activation	IL-4, IL-10, TGF-β, IGF-1, BDNF; anti-inflammatory STAT signaling	Suh et al., 2013; Tang and Le, 2016; Kwon and Koh, 2020
Trophic factors	Reduced or absent production of neurotrophic factors	Increased secretion of BDNF, GDNF, CNTF, and VEGF	Reduced production of neurotrophic factors	Increased secretion of BDNF, IGF-1 and GDNF	Suh et al., 2013; Badia-Soteras et al., 2025
Functional effects on neurons	Promote neuronal damage and cell death	Support neuronal survival, synaptic plasticity, and repair	Promote beurotoxicity and chronic inflammatory responses	Support neuronal regeneration, synaptic remodeling, and tissue homeostasis	Choi et al., 2014; Liddelow et al., 2017
Role in neuroinflammatory disease	Associated with chronic neuroinflammation (Alzheimer’s disease, Parkinson’s disease, ALS, multiple sclerosis)	Promote neuronal damage in neurodegenerativediseases (Alzheimer’s disease, axonal injury)	Contribute to chronic neuroinflammation and neuronal damage (multiple sclerosis, Alzheimer’s disease and axonal injury)	Associated with anti-inflammatory responses, early neuroprotection, and tissue repair	Tang and Le, 2016; Liddelow et al., 2017

The A1/A2 and M1/M2 terminology is used here as a heuristic and historically established framework to summarize prototypical glial programs. Increasing evidence indicates that astrocytes and microglia exhibit dynamic, context-dependent, and overlapping activation states that exist along multidimensional continua rather than as discrete or fixed phenotypes.

## 5 Discussion

### 5.1 Functional implications of astrocyte–microglia crosstalk

Astrocytes and microglia engage in dynamic, bidirectional communication that orchestrates CNS development, homeostasis, and response to injury. This crosstalk is mediated by an integrated set of soluble factors, extracellular vesicles, purinergic messengers, and direct cell–cell interactions, collectively shaping neuronal connectivity, immune surveillance, and tissue repair (Delpech et al., 2019; Matejuk and Ransohoff, 2020; Rostami et al., 2021). Rather than acting in isolation, coordinated astrocyte–microglia signaling drives activity-dependent remodeling of neuronal networks and preservation of CNS stability (Dzyubenko and Hermann, 2023; Badia-Soteras et al., 2025).

At the molecular level, these interactions converge on shared intracellular signaling hubs, including NF-κB, JAK/STAT3, MAPK/ERK, and TGF-β/Smad pathways. NF-κB activation signaling promotes pro-inflammatory crosstalk by driving the coordinated release of IL-1β, TNF-α, and IL-6, thereby amplifying microglial reactivity and astrocytic inflammatory responses (Hein et al., 2026). In contrast, activation of STAT3 and Smad2/3 pathways favors anti-inflammatory and neuroprotective programs, that support tissue repair, synaptic stabilization, and resolution of inflammation (Liu et al., 2015; Vidovic and Spittau, 2024).

The functional relevance of this molecular integration becomes evident in pathological contexts. Disruption of astrocyte–microglia communication contributes to sustained neuroinflammation and progressive neuronal dysfunction in neurodegenerative disorders such as Alzheimer’s disease, Parkinson’s disease, amyotrophic lateral sclerosis, and multiple sclerosis (Sochocka et al., 2017; Wu and Eisel, 2023). In these conditions, maladaptive glial signaling can lock astrocytes and microglia into self-reinforcing inflammatory states, exacerbating synaptic loss, demyelination, and neuronal death, as revealed by recent transcriptomic analyses of glial activation states in neurodegenerative disease (Leng et al., 2023; Li et al., 2023).

Conversely, preservation or restoration of coordinated glial signaling promotes neuroprotection through enhanced phagocytic clearance, metabolic support, and the release of neurotrophic factors such as IGF-1, BDNF, and NGF (Rothhammer et al., 2018; Theophanous et al., 2024). Purinergic signaling and extracellular vesicle–mediated communication further act as rapid, spatially precise modulators that enable astrocytes and microglia to sense subtle changes in the neural microenvironment and adjust their responses accordingly (Matejuk and Ransohoff, 2020; Gao et al., 2023).

Collectively, these findings highlight astrocyte–microglia crosstalk as an integrated molecular signaling network that critically determines whether neuroinflammatory responses remain protective or become detrimental. Understanding how these signaling networks are temporally and spatially coordinated is therefore central to identifying therapeutic strategies aimed at restoring CNS homeostasis.

### 5.2 Human-based models to dissect glial signaling networks

Although much of our current understanding of astrocyte–microglia communication originates from rodent studies, increasing evidence indicates that species-specific differences in gene expression, immune responsiveness, and signaling dynamics constrain their translational relevance. Transcriptomic analyses have shown that only about 30% of human astrocyte-enriched genes are enriched in mice and that human astrocytes display distinct mitochondrial resting-state respiration and greater susceptibility to oxidative stress than mouse astrocytes (Batiuk et al., 2020). Likewise, single-cell and bulk transcriptomic studies in human brain tissue have revealed marked species-specific differences in astrocytic and microglial responses to pathological protein clearance and inflammation, including divergent expression of Alzheimer’s disease risk genes such as CLU, MEF2C, APOE, and PILRA (Smith et al., 2022). Together, these findings underscore the need for human-based experimental models to interrogate glial signaling in physiologically and pathologically relevant settings.

Human induced pluripotent stem cell (iPSC)–derived astrocytes and microglia provide a tractable platform for reconstructing human-specific inflammatory and neurotrophic signaling pathways under controlled conditions. Recent work using human iPSC-derived astrocytes has revealed disease-associated alterations in glial phenotypes. For instance, astrocytes carrying the Alzheimer’s disease–associated APOE4 genotype adopt a senescent and pro-inflammatory state that compromises neuronal support, highlighting the utility of human stem cell–derived models for studying pathological glial signaling (Cáceres-Palomo et al., 2025). In addition, co-culture systems using iPSC-derived glial cells suggest that cytokine responsiveness and inflammatory signaling are not fully conserved across species, supporting the view that key pathways such as NF-κB and JAK/STAT may be engaged differently in human and rodent glia (Batiuk et al., 2020; Smith et al., 2022). These platforms allow precise manipulation of genetic and environmental variables, enabling the study of disease-associated glial phenotypes and patient-specific responses. Thus, human-based models do not simply reproduce rodent findings in a human cellular background, but can reveal divergent inflammatory thresholds, disease-risk gene responses, and pathway engagement patterns that may alter therapeutic interpretation.

Beyond reductionist co-culture approaches, three-dimensional brain organoids and organ-on-chip systems provide multicellular environments in which astrocyte–microglia communication can be studied within a more physiologically relevant tissue architecture. Incorporation of microglia into brain organoids influences neuronal maturation, synaptic pruning, and inflammatory responses, highlighting the importance of spatial context in glial signaling (Sabate-Soler et al., 2022; Mrza et al., 2024). Organ-on-chip platforms further refine these models by introducing controlled perfusion, mechanical cues, and compartmentalization, thereby enabling real-time analysis of cytokine gradients, extracellular vesicle exchange, and neurovascular interactions (Yoon et al., 2021; Wu et al., 2024; Di Stefano et al., 2025).

Crucially, the value of these human-based models lies not only in their technical sophistication, but also in their ability to capture human-relevant molecular signaling dynamics that are difficult to resolve in animal systems. By enabling the study of temporal signaling coordination, pathway convergence, and context-dependent glial responses, these platforms help bridge the gap between simplified in vitro systems and the complexity of the human brain microenvironment, thereby accelerating the translation of glia-targeted therapeutic strategies.

### 5.3 Translational applications and therapeutic perspectives

The growing understanding of astrocyte–microglia communication has opened new avenues for translational research aimed at modulating neuroinflammatory processes in a context-dependent manner. Rather than indiscriminately suppressing inflammation, emerging therapeutic approaches aim to shift astrocyte and microglial activation toward neuroprotective and reparative phenotypes while preserving essential immune functions through modulation of pathways such as TGF-β/Smad and STAT3 signaling (Rothhammer et al., 2018; Zhang et al., 2021; Vidovic and Spittau, 2024).

Recent evidence suggests that metabolic and signaling reprogramming of microglia may represent important determinants of disease progression linking cellular metabolism to inflammatory and neurotoxic phenotypes (Sadeghdoust et al., 2024). Experimental evidence indicates that pro-inflammatory microglial activation is frequently associated with a metabolic shift from oxidative phosphorylation to glycolysis, whereas oxidative metabolic programs are linked to reparative and neuroprotective functions (Baik et al., 2019). Within this framework, astrocyte–microglia interactions emerge as attractive therapeutic targets, as they integrate growth factor signaling (NGF, BDNF, IGF-1), cytokine networks, and intracellular pathways such as STAT3, NF-κB, and Smad2/3 that collectively govern glial plasticity. Experimental studies have demonstrated that selective modulation of these pathways can promote anti-inflammatory and neuroprotective responses while preserving essential immune functions, particularly through TGF-β/Smad-mediated regulation of astrocyte reactivity and microglial activation states (Rothhammer et al., 2018; Zhang et al., 2021).

Importantly, the dual role of astrocytes and microglia as both protectors and potential drivers of pathology underscores the necessity of reprogramming glial activation states rather than blocking inflammatory signaling altogether. Experimental studies have shown that glial-derived TGF-β and IGF-1 can promote resolution of inflammation and enhance tissue repair, illustrating how selective pathway modulation biases glial responses toward neuroprotective outcomes (Suh et al., 2013; Luo et al., 2024; Zhang et al., 2021).

Humanized experimental platforms, including iPSC-derived glial cultures, brain organoids, and organ-on-chip systems, provide unprecedented opportunities to define the temporal boundaries and molecular determinants of this therapeutic window. By capturing human-specific signaling dynamics and disease-relevant glial phenotypes, these models can guide the development of precision therapies aimed at restoring glial homeostasis while minimizing off-target effects and immunosuppression.

### 5.4 Limitations and future directions

Despite significant advances in glial biology, much of our current understanding of astrocyte–microglia interactions still derive from rodent models, which do not fully recapitulate the complexity of human astrocyte and microglial function. These systems often fail to capture species-specific differences in gene expression, immune responsiveness, and signaling dynamics, limiting their translational relevance. Consequently, there is a pressing need for studies employing human-derived cells and physiologically relevant experimental platforms to bridge this gap and enhance the clinical applicability of basic research findings.

Beyond species differences, an additional limitation of many existing studies is their reliance on static or short-term experimental designs. Astrocyte and microglial phenotypes are highly dynamic and evolve over time in response to developmental cues, injury, and disease progression. Longitudinal studies, capable of tracking glial states across defined temporal windows, will be essential to understand how astrocyte–microglia communication shifts from adaptive to maladaptive signaling and to identify critical periods for therapeutic intervention.

In this context, a promising emerging frontier is the concept of innate immune memory in glial cells. Recent evidence indicates that innate immune training can epigenetically reprogram CNS-resident myeloid cells toward pro-reparative phenotypes, promoting remyelination and functional recovery following demyelinating injury (Tiwari et al., 2024). These findings raise the possibility that glial cells may retain memory-like inflammatory states that shape subsequent responses and contribute to the persistence of neuroinflammation in chronic CNS disorders. Elucidating the molecular basis and long-term consequences of this memory-like behavior will require experimental approaches that integrate temporal resolution with molecular depth.

Future progress will also depend on the integration of multi-omic and spatially resolved technologies, including single-cell RNA sequencing, spatial transcriptomics, epigenomic profiling, and proteomics. These approaches are critical for resolving glial heterogeneity, mapping cell–cell communication networks and linking molecular signatures to functional phenotypes within intact tissue contexts. For example, spatial transcriptomic analyses of the amyloid plaque niche have revealed microglia–astrocyte crosstalk, heterogeneous plaque-associated glial responses, and altered neuronal signaling in Alzheimer’s disease models (Mallach et al., 2024). When combined with human iPSC-derived models, brain organoids, and organ-on-chip platforms, multi-omic strategies will enable a systems-level understanding of astrocyte–microglia signaling across space and time.

Together, integrating molecular signaling, temporal dynamics, and human-relevant experimental models will be essential to unravel the complexity of astrocyte–microglia interactions and to develop precision strategies capable of reprogramming glial states toward sustained neuroprotection without compromising essential immune functions.
